# Glances at the Hospitals

**Published:** 1900-10-13

**Authors:** 


### GLANCES AT THE HOSPITALS.

### THE CORNWALL INFIRMARY, TRURO.

The receipts amounted to £1,801, and the expenditure to £1,900. A sum of £260 was obtained by the sale of Indian 3 per cent. stock. The cost per bed was £61 6s., and the average stay in the hospital of each patient was 27 days. It is proposed to make considerable alterations in the hospital, and for this purpose the sum of £3,500 will be required; but the committee do not recommend the work to be done immediately unless funds are forthcoming. The improvement of the operating theatre, being urgent, is to be proceeded with. The report runs from May 31st, 1898 to May 31st, 1899. This is opposed to the custom of the majority of infirmaries, and there seems to be no good reason why the usual dates should not be used. On the beginning of this year the hospital contained 29 patients, and 416 were admitted during the 12 months, making a total of 445. Of these 261 were discharged cured; 121 relieved; 19 not relieved; 8 for other reasons; 18 died; and 18 remained under treatment. The medical and surgical tables are incomplete.

### THE BLACKBURN INFIRMARY.

The total net receipts from all sources amounted to £7,475 for the year 1899, being an increase of £232 on those of the previous year. The expenditure on the ordinary fund amounted to £6,143. A sum of £1,000 has been transferred to the building fund, and £156 has been spent on repairs. There is a balance in hand of £978. Manifestly therefore the funds of the hospital are in a sound condition. A new wing, being the Blackburn Memorial of the Diamond Jubilee, is being erected at a cost of £10,000, of which £7,073 has already been received. Neither the cost per bed occupied, nor the cost per patient is given. On January 1st there were 90 patients in residence; and 1,522 were admitted during the year. The average stay in the hospital was twenty-seven days, and the death-rate was 5·89 per cent., or, excluding those who died within forty-eight hours of admission, 3·76 per cent. Of the total number of in-patients 824 were discharged recovered, 235 relieved, and 72 died: 92 patients were sent to various convalescent homes. The out-patients numbered 4,671. The medical and surgical tables show the numbers under treatment for the various diseases and also the numbers of those who recovered, who were relieved, and who died. The table of operations is prepared on the same lines. Altogether 412 operations were performed on the in-patients, and death followed in 15 cases. There is no table showing the nature of the anæsthetics administered—a regrettable omission.

### DERBYSHIRE ROYAL INFIRMARY.

Here the total ordinary receipts amounted to £7,778, and the total ordinary expenditure to £8,617, showing a deficiency of £838. The extraordinary receipts reached the respectable sum of £3,157, and the extraordinary expenditure, including the adverse balance of £838, to £1,515, thus permitting £1,641 to be placed to the capital account. The daily average number of in-patients was 147, and the average stay of the patients was 26½ days. At the end of last year there were 149 patients in the hospital, and 1,830 were admitted during the year, making a total of 1,979. Of this number 1,252 were cured, 38 were made out-patients, 279 relieved, 88 for other reasons, 155 died, and 147 remained under treatment. The year runs from September 28, 1898, to September 28, 1899, and there is a misprint in the table on page 32. The medical and surgical tables are not well compiled, as they merely show the number of patients under treatment for the various diseases. The table of operations is better arranged, but even here the "cured, relieved, &c.," are placed under one column. There was a total of 907 operations and only 21 subsequent deaths. We notice that the operation for the radical cure of hernia was performed 22 times without a death, and that for strangulated hernia 13 times with 6 deaths. Here the high mortality was doubtless owing to delay in the patients to the hospital. Intra-abdominal operations were performed in 34 cases, with 7 deaths. A table showing the anæsthetic used in these 907 operations would have been both interesting and instructive; and we hope the house surgeon will insert one in the next annual report.

### CHELTENHAM GENERAL HOSPITAL.

The total hospital receipts amounted to £5,210, and there was an excess of expenditure over income of £1,559; but this deficit has been in great part caused by structural alterations which came to £800. The average cost of each in-patient was £4 14s. On the first day of January, 1899, the hospital contained 59 patients and 745 were admitted during the year. Of these 366 were discharged cured, 182 relieved, 13 not relieved, 22 were discharged for other reasons, 40 died, and 45 remained under treatment. The out-patients cost 3s. 5½d. per head, and 4,771 were treated during the year, and 327 were visited at their own homes. The medical and surgical tables are carefully compiled. There were 162 operations performed, and only three deaths subsequently took place. Attached to the hospital is a branch dispensary. Here 3,835 cases were treated. In addition we learn that 1,634 dental cases were attended to, and we thus have a grand total of 11,044 patients treated during one year in this charity. As the population of Cheltenham is only 44,000, we have one in every four of the population receiving gratuitous medical attendance; and in this calculation the other charitable hospitals and the Union Infirmary are not included.

### THE PRINCE ALFRED HOSPITAL, SYDNEY.

The income for the year 1899 was £15,704, of which Government contributed £9,536. This account shows that on the last day of 1898 there was a deficit of £958, and that on the last day of 1899 this had increased to £2,102. The average cost per bed occupied was £69 16s., and this sum has been obtained after deducting the cost of the out-patients' department and also the cost of buildings and permanent improvements. Contributions from patients amounted to £2,144. The maintenance of sick paupers is put down at £6,361, and in addition there was a Government subsidy of £3,175. As regards the nursing staff and the number of women anxious to be trained, much the same condition of affairs seems to prevail in Australia as in England, for we read that there were 303 applications and only nineteen vacancies. During the year 3,517 patients were admitted. The medical and surgical tables are admirably arranged. Opposite the name of the diseases are shown the number of patients treated; the numbers cured, relieved, and died; and in addition to this there is a general summary of the operations and the results thereof. Indeed, we only miss a table showing the nature of the anæsthetic used. A medical school is attached to the hospital.

### PRESTON ROYAL INFIRMARY.

The income for 1899 amounted to £5,989. The maintenance account shows a balance of £565, and the former deficiency on this account has now been reduced to £260. The collections in mills and workshops amounted to £2,098, and £351 was received for the maintenance of in-patients. It is proposed to spend £22,500 on adding to and improving the hospital. The Diamond Jubilee Fund amounts to £8,000, and Dr. R. C. Brown has undertaken to defray the cost of the new operating theatre at a cost of £2,700; but it is to be hoped that the committee will not spend more than they have actually in hand, as a building debt is sure to cramp the usefulness of any hospital. About £13,000 will be required, and an appeal for this sum will be made when fuller details of the cost can be submitted. There can hardly be a doubt that the inhabitants of Preston and of the country generally will speedily subscribe what the Governors ask for. The total number of patients treated during 1899 was 5,196. Of these 933 were in-patients; and 4,263 were out-patients, or Home department cases. The tables are carefully drawn out, and the results of treatment easy to follow; but we miss, again, a table showing the anæsthetic used in the 171 major operations.

### THE COTTAGE HOSPITAL, NORTH ORMESBY.

The total receipts for 1899 were £3,741, and the ordinary expenditure was £3,398, showing a balance to the good of £343, which is very satisfactory in so small a hospital; but the Board points out that the finances are not really so favourable as they seem, because they have opened a new wing, and this will increase the expenditure.

The statistics show the following movements of patients. On January 1st 55 patients were in the hospital; 572 new cases were admitted, making a total of 627. Of these 509 were discharged cured, 37 relieved, 24 died, 5 were discharged for various reasons, and 52 remained under treatment. The average duration of residence was 31 days. A complete list of the diseases from which the patients suffered and of the operations performed is given, but the results should have been shown in parallel columns. Certainly a table of the deaths is given: but this is hardly sufficient, and at its best it necessitates reference from one table to another. The medical table shows that 16 cases of pneumonia were under treatment, and the death table shows that 8 patients died of this disease, but we get no explanation of this high mortality. We should also like to be informed what anæsthetics were used in the 106 operations.

### GENERAL INFIRMARY, WORCESTER.

The income for the year, excluding legacies, but including donations, was £4,869, and the expenditure was £7,564, showing a deficiency of income of £2,695; but the year's legacies amounted to £1,590, and upwards of £59,000 are invested.

On the first day of 1899 there were 89 patients in residence and 1,124 were admitted during the year. Of these 743 were discharged cured, 272 were relieved, 28 were discharged not relieved, and 79 died. At the end of the year 91 remained in the hospital. The total number of out-patients was 5,692. If dental cases be included we have a total of 7,950 for the year 1899; and this again shows that something like one in every five of the population of Worcester received gratuitous medical advice in this single hospital.

One of the tables shows, in parallel columns, the diseases under treatment and the results; but this admirable plan is not carried out in the table of operations, and we failed to find any record of the anæsthetics used.

